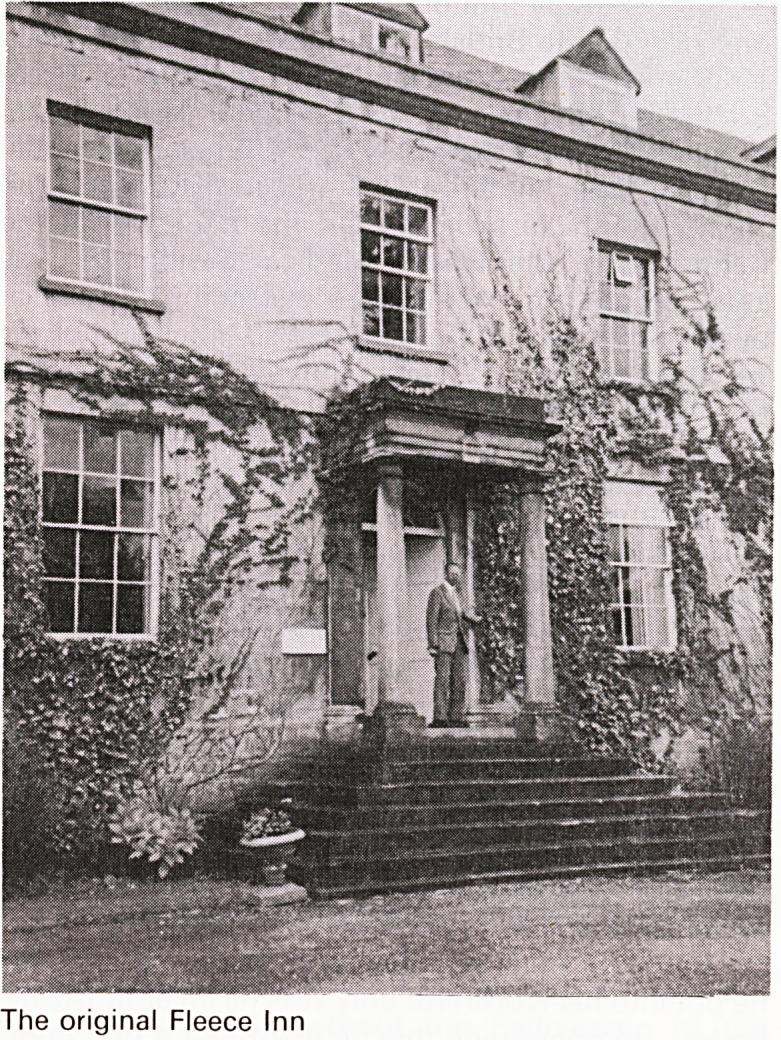# The Fleece Medical Society

**Published:** 1983

**Authors:** H. J. Eastes

**Affiliations:** General Practitioner, Marshfield


### The Fleece Medical Society

**H. J. Eastes, M.B., B.S., F.R.C.G.P.**
General Practitioner, Marshfield

The Bristol Medico-Chirurgical Society was formed in 1874 – and 9 years later – a hundred years ago – its journal first appeared. However, some 84 years earlier, on 21st May 1788, five members of our profession – all school friends or fellow students – met in the parlour of the Fleece Inn at Rodborough, in the valley between Stroud and Nailsworth and resolved to set up the Gloucestershire Medical Society, better known as the Fleece Medical Society.

*The Fleece Inn*, built in 1753, was patronised by members of the woollen trade which flourished in the valley. In 1853 the licence seems to have been transferred to a building nearer Stroud and known as the Old Fleece Inn. The original building in which Jenner and his friends met is now a private residence – Hillgrove House on the A46 (Figure 1).

Two of the five members founding the Fleece Medical Society, Edward Jenner of Berkeley and John Hickes of Gloucester, were already members of the Convivio-Medical Society meeting at the Ship Inn at Alveston near Bristol, a largely social club whose members were threatening to expel Jenner for his insistence on the importance of Coxpox as a protection against Smallpox. This may have been the spur that brought these five friends together – to form what was, I believe, the oldest provincial medical society of which records still exist. The minutes are preserved, thanks to Sir William Osler who bought them from Dr. Alfred Henry Carter of Birmingham and later bequeathed them to the Royal College of Physicians in whose library they have been since 1928.

The original Fleece Inn

*The Rules of the Society* were as follows:

(1) Though first instituted and already twice met for conviviality that it be for the future extended to the more important end of improvement in the different branches of science connected with Medicine and Surgery.

(2) That it meet three times a year on the first Wednesday in June, the last in July and the second in September.

(3) That its first meeting be on 30th July next at the Fleece Inn.

(4) That each member shall contribute half a guinea annually to defray any incidental expenses.

(5) That members endeavour to provide original papers on medical and allied subjects. The papers to be discussed with candour yet freedom and fair copies on folio foolscap paper with inner blank margins of at least one inch be delivered to the President at the first or second meeting after that at which read – in default of which – shall forfeit half a guinea to common stock.

(6) That as the members have in view the extension of useful knowledge which cannot be effected by their meetings in any other way than by publishing their papers; when a sufficient number of these shall have been communicated they shall severally be received by a committee consisting of the whole Society except respective authors. Decision of the Society to be final on the merit of the paper, though the author shall still have it in his power to refuse its publication.

(7) That when a selection has thus been made the papers shall be redelivered to their authors for three months who after correcting and amending shall under penalty of half a guinea for each paper restore them to the President.

(8) That they shall be published at the expense of and for the benefit of the Society.

(9) That each volume so published shall be dedicated by the President by rotation to such a person or persons as he shall choose and the Society shall not disapprove.
(10) That the chair be taken precisely at 1 o'clock in the afternoon, and that each member not present at that hour shall forfeit half a guinea to common stock.
(11) That each member who neglects entirely to attend shall forfeit one guinea to common stock.
(12) That the reading of papers and medical conversations are to continue till four o'clock and that a dinner at 3/6 a head, exclusive of liquors, be on the table at a quarter past four o'clock. The ordinary to be paid by all members present or absent and the wines and other subsequent expenses by those present.
(13) That as the principal part of the view of this Society is the mutual communication of knowledge without fear, reserve or any other authority than that of truth, no new member shall be admitted for these seven years to come whose age exceeds forty years.
(14) That nevertheless as it is still wished to retain that conviviality for the sake of which the Society was first instituted it shall be of the most select kind consisting at present of the following old friends, school fellows and fellow students.

1. John Heathfield Hickes M.D. of Gloucester (37)
2. Edward Jenner, Surgeon of Berkeley (39)
3. Thomas Peytherus, Surgeon of Ross (35–38)
4. Daniel Ludlow, Jr., Surgeon of Sodbury (31)
5. Caleb Hiller Hillier Parry M.D. of Bath (33)

(15) That for the same reason no two physicians or surgeons from the same place shall become members.
(16) That for the same reason the mode of admitting members be by the unanimous application of the Society to such a person or persons as they may wish to have among their number.
(17) That no visitors be admitted.
(18) That each member shall in rotation for three meetings hold the office of President, Secretary and Treasurer, regulating the conversations, making minutes of the proceedings of the respective meetings and in delivering in an annual account of the funds of the Society.
(19) That Dr. C. H. Parry be appointed President of the three meetings next ensuing. We whose names are underwritten approve and declare ourselves bound by the above regulations.

Certainly a very select society.

*John Heathfield Hickes*, born at Pedington Manor just outside Berkeley, must have known Jenner during childhood. Both went to Rev. Washbourne's Grammar School at Cirencester where they met Parry. Both Hickes and Jenner proceeded to Edinburgh Medical School, part of the time together. Hickes qualified M.D. in 1776 and settled in Gloucester where he was on the staff of Gloucester County Hospital for 14 years. During this time he carried out investigations into swine pox and reported his findings to the Fleece M.S. in 1790. Hickes subsequently moved to Bristol and by 1799 Parry refers to Dr. Hickes of Bristol and says he has been his friend for 40 years. Finally he returned to his native Pedington where he died in 1808.

*Edward Jenner* was the third son of the Vicar of Berkeley and was born on 17th May 1749. When quite young he evinced a taste for natural history in forming a collection of nests of the dormouse. He also accumulated a fine collection of fossils which at a later date he catalogued. Among his many accomplishments he played the violin and the flute, wrote poetry and composed ballads. He was apprenticed for 6 years to Daniel Ludlow, Sr., surgeon of Sodbury – 'a person eminent in his profession;' and then in 1770, at the age of 21, became the resident pupil in London, of John Hunter, a man twice his age – anatomist, surgeon to St. George's Hospital and owner of a menagerie at Brompton. They became staunch friends and Jenner availed himself of all the opportunities this appointment offered. He became an expert in dissection and the display of 'most delicate organs'. A particularly fine collection of these tracing the development of the ovum of the domestic fowl from the earliest stages to its completion he bequeathed to his friend and biographer Dr. Baron of Gloucester. Hunter encouraged his interest in natural history and repeatedly advised him to experiment. He studied the hibernation of animals and the strange breeding habits of the cuckoo. His account of the latter subject was published in the Philosophical Transactions of the Royal Society in 1788. In that year he set up practice in Berkeley and married Catherine Kingscote. In 1792 he was made M.D. in St. Andrews. In 1794 he developed typhus from which he nearly died. He was attended by his friend Parry.

Since his apprenticeship days he had been interested in the West Country belief that an attack of Cowpox gave protection against Smallpox. In 1796 an outbreak of Cowpox locally gave him the opportunity to experiment. On 14th May he inoculated the boy James Phipps with lymph from the Cowpox vesicle on the finger of the dairymaid Sarah Nelmes and on 1st July he inoculated Phipps with lymph from a Smallpox vesicle. It did not take. The following year he treated more cases in the same way with the same result and so he decided to publish. He submitted his paper to the Council of the Royal Society and received back 'a friendly admonition that as he had gained some reputation by his former paper to the Royal Society (on Cuckoos) it was advisable not to present this – lest it should impair his established credit'. In June 1798, after a further score of cases he published 'An inquiry into the causes and effect of the Variolae Vaccinee' and dedicated the paper to his friend Parry.

Jenner's message was readily received in America and most of Europe but only slowly in England.

Continuing his studies of natural history he turned his attention to the migration of birds but while writing his paper on the subject he died of apoplexy on 26th January 1823. His unfinished paper was presented to the Royal Society later that year.

*Thomas Paytherus*, a surgeon of Gloucester, was a few years younger than Jenner. He would have known Hickes, Physician at the County Hospital and Jenner who visited from time to time. Paytherus moved to Ross in 1783 and in 1786 when a case of angina died suddenly he got Jenner and Hickes to come to the postmortem. Jenner wagered the coronaries would be ossified – remembering a case of his own several years before and quoted by Parry in his publication on the subject.

Paytherus moved to London in 1797 and in 1801 published 'A comparative statement of facts and observations relative to the Cowpox'. In 1828 he retired to Abergavenny.

*Daniel Ludlow Jr.* became friends with Jenner while he was apprenticed to his father at Sodbury. Like Jenner he was a keen student of natural history and again like Jenner was apprenticed to John Hunter for a while. He joined the company of surgeons in 1783 and first practised at Sodbury and later moved to Bath and Corsham. He died of tetanus in 1802, probably attended by his friend Parry.

*Caleb Hillier Parry* (1755–1822), born at Cirencester, the eldest of 10 children of Joshua Parry, a Presbyterian divine, 'an excellent classical, Welsh and Hebrew scholar'. Coming from a Pembrokeshire family with considerable estates near Narberth, Joshua, one of 21 children, left Llangan for Cirencester where he died in 1776. Caleb's mother, daughter of Caleb Hillier – after whom her first born was named, inherited properties at Upcott and Minety, some 60 and 10 miles from Cirencester respectively. Educated first at Rev. Washbourn's Grammar School with Jenner and Hickes and then at the famous Dissenter's Academy at Warrington where he first met the beautiful Miss Rigby, later to become his wife.

In 1773 he went to Edinburgh University under Mr. Cullen moving to London to live with Dr. Thomas Denman – physician – accoucheur to the Middlesex Hospital. He returned to Edinburgh in 1777 and graduated M.D. the following year with a thesis on Rabies Contagiosa dedicated to Lord Bathurst, his father's friend and patron. While at Edinburgh he had become President of their Medical Society and was largely instrumental in obtaining its Royal Charter.

In October 1778 he married the beautiful and accomplished Miss Rigby and after travelling in Holland, Flanders and France he settled in Bath in November 1779 and scarcely left it for more than a day for the rest of his life.

Georgian Bath was at the height of its fame. Beau Nash, who had established it as the fashionable resort, had been dead 18 years. The two Woods had built South Parade, Queen's Square, The Circus, Royal Crescent and the Assembly Rooms. The following year the Pump Room was rebuilt on a large scale. Mozart was at the height of his fame – Beethoven was about to compose his masterpieces and in a few years Napoleon would be the focal point of European history. What a time for this remarkably able and handsome man to set up practice. He had much dignity of manner and a certain playfulness – always ready with an amusing anecdote or appropriate allusion.

Parry first settled at 13 Catherine Place but soon moved to 27 The Circus and continued to practice from there for the rest of his active professional life, only moving to the house he built in Somerfield Park – Sion Place after his hemiplegic attack in 1816. In 1925 a plaque was placed on 27 The Circus recording this as 'The home of Caleb Hillier Parry and afterwards his youngest son, Sir Edward Parry, Arctic Explorer'.

To Parry success came slowly. His earnings for his first year are recorded as £39.19.0 and for his second £70. Even by the fifth year only £239 but by the tenth £1600 and later over £3000. Over the years many of his poorer patients were charged no fee. That the buildup was slow was fortunate for posterity as it gave him time for careful note-taking and dissection and the cultivation of his other talents. In 1881 he published 'Proposal for a history of the fossils of Gloucestershire' of which he had a considerable collection. He was appointed physician to the 'Puerperal Charity' and next year a governor and physician to the Bath General Hospital (founded 1742) and later known as the Royal Mineral Water Hospital.

Already interested in agriculture, in 1787 he took a farm within walking distance of The Circus and started sheep rearing. In July 1788 he read a paper at the first meeting of the Fleece Medical Society describing a case of Angina with dissection. In September that year he was admitted licenciate of the Royal College of Physicians – equivalent to the M.R.C.P. these days.

Soon he was appointed physician to the new Casualty Hospital which in 1826 was merged with the City Hospital to become the Royal United Hospital to which Parry's eldest son, Charles Parry was the first physician.

During the next quarter of a century the daily list of his patients included 'nearly the whole catalogue of British nobility and many of the most distinguished men in the Kingdom', and correspondence preserved testifies the benefits they received as a result of his attentions. He formed friendships and kept up correspondence with many of these including Sir William Herschel, discoverer of the planet Uranus, Admiral Lord Rodney, Sir Joseph Banks, Lord Somerville, Edmund Burke and William Windham. In 1792 he published a paper on 'The effects of compression of the arteries in various diseases, particularly in those of the head', in which he discussed his observations on the effect of compression of the carotids on convulsions and hemicrania and of the digital arteries in whitlow. He discovered the effect of pressure on the carotid sinus in slowing the heart.

After John Hunter's death he published 'An inquiry into the symptoms and causes of Syncope Anginosa – commonly called Angina Pectoris – illustrated by dissections'. In the view of Sir Thomas Lewis it was Parry who first realised it was the inadequacy of the coronary circulation that was the fundamental factor in Angina.

In 1814 he published 'Cases of Tetanus and Rabies Contagiosa or Canine Hydrophobia', dedicated to Edward Jenner. He suggested Hydrophobia could be prevented by enforcing a tax on dogs with power to confine dogs suspected to be mad. It was some 70 years later that such legislation was introduced and that together with the muzzling order virtually eradicated the disease. At last in 1815 he produced the first half of the work he had so long contemplated, 'Elements of Pathology and Therapeutics', which soon went into a second edition.

His most original work he reprinted a year later – 'Experimental Inquiry into the nature, causes and variety of the arterial pulse and into certain other properties of the large vessels in animals with warm blood', dedicated to Sir Joseph Banks. It was based on operations mainly on sheep and rabbits, showing that the heart's systole was the real cause of the pulse. He also demonstrated the anastomosis of vessels and the regeneration of the carotid artery in sheep after its removal.

On 25th October 1816 a right hemiplegia developed and rendered his speech almost unintelligible and in April 1817 he moved to his new house in Somerville Park where he directed his still not inconsiderable energies to running his garden and his farm. He read extensively and dictated to his daughters various works including an essay on the character of Hamlet.

This remarkable doctor was also one of the most scientific agriculturalists of his day and received distinctions from many societies. His non-medical publications include articles on English Rhubarb, Wild Endive, A Prize crop of cabbages, Causes of decay in wood, Obervations on the crossing of animals, On the English Race Horse and many on Merino sheep and Wool.

He was instrumental in proving that we could produce even better wool than Spain and in 1800 George III accepted a piece of blue cloth manufactured from Dr. Parry's wool and declared that from its excellent quality he should feel a pride in wearing it.

On Saturday, 9th March 1822 this most gifted and remarkable man died $5\frac{1}{2}$ years after his first stroke. The family intended there should be a private funeral but on 11th March a letter signed by 39 of his colleagues was sent to Dr. C. H. Parry entreating that 'we be permitted to attend it'.

The Bath Chronicle of Thursday, 21st March 1822 reported on the funeral of the late Caleb Hillier Parry M.D., F.R.S. etc. which gave rise to scenes such as had never before been seen in Bath. The hearse was followed by fourteen mourning coaches each drawn by four horses followed by half the same number of gentlemen's coaches of the family and distinguished friends. Vast crowds lined the streets and filled every window. Dr. Jenner was one of the pall bearers. He was interred in the Abbey and his colleagues caused a tablet to be raised to his memory.

In 1825 Parry's son published 'Collections from the unpublished writings of the late Caleb Hillier Parry' which was to have formed the basis of his second volume on Therapeutics. The preface is a philosophical dissertation on the nature of human knowledge and the means of attaining it. There follow many careful case notes and dissections. There is an interesting description of aneurysmal dilatation of the auricles with clots in a case of mitral stenosis with dropsy treated with squills and digitalis. Sir Thomas Lewis considered Parry's treatment of heart failure was ahead of his time.

Most interesting are the pages describing enlargement of the thyroid gland in connection with enlargement or palpitations of the heart. He describes 5 cases – the first observed in August 1786 and this preceeds by many years those of R. J. Graves of Dublin in 1835.

Sir William Osler, speaking at the Royal Mineral Water Hospital at Bath in 1914, said that if any name is to be associated with Exophthalmic Goitre it should be Parry's—a view also held by Sir Thomas Lewis and by George Armitage the Leeds thyroid surgeon (7).

Parry had accumulated a fine library containing some 500 books on medicine and allied subjects and his son, Charles Henry Parry M.D., gave them to the Royal United Hospital. They are now to be found in the Medical Library of Bristol University. There are volumes by Bell, Eustachius, Galen, Hippocrates, Morgagni, Priestly, Scarpa, Sydenham and many others.

I have told you more about the members of the Fleece Medical Society than about the Society itself. Two of its members were F.R.S. (as were two of Parry's sons). At its first meeting Jenner presented a case of Mitral Stenosis with dissection and related to acute Rheumatic Fever and Parry described a case of his with Angina Pectoris and demonstrated inadequacies in the coronary arteries. Much of the work leading up to Jenner's publication on Variolae Vaccinae was presented to the Society by Jenner and Hickes. Jenner reported on a case of Hydatid of the Kidney 'successfully treated with Oil of Turpentine' on 28th July 1790, also the effects of calomel in relieving suppression of urine and the case of a boy 'passing extraneous substance into the bladder'.

The Society met on Wednesday, 5th June 1793 with Parry in the chair. Papers were read on Puerperal Fever, Dropsy, Children's Palsy and Lice. This was their last recorded meeting, Why these meetings then came to an end we have no evidence. In spite of their obvious intention to publish as a Society there is no evidence that they achieved it. I think they had made it too difficult for themselves with their rules. Parry was certainly getting very busy. We know that John Hunter's Angina had been an inhibiting factor and perhaps Jenner's dramatic attack of Typhus had something to do with the sudden end of this ambitious early Medical Society.

### REFERENCES

1. Minutes of the Gloucestershire Medical Society, Library of the Royal College of Physicians, London.
2. *The Bath Chronicle*, 21st March 1822.
3. PARRY, C. H., Collections of unpublished writings of Caleb Hillier Parry. London, 1825. Vols. 1 and 2.
4. Lives of British Physicians. 1830, p. 301.
5. Records of an Old Medical Society – some unpublished manuscripts of Edward Jenner. *B.M.J.* 1896, **1**, 1296–8.
6. Caleb Hillier Parry M.D., F.R.S., 1755–1822. A great Welsh Physician and Scientist. *Proceedings of the Cardiff Medical Society*, 1940–41.
7. ARMITAGE, G. (1956). Parry's Disease. *Leeds Medical Journal* **5**, 9–24.
8. PROUDFIT, W. L. (1981) *British Heart Journal* **465**, 89–94

## Figures and Tables

**Figure f1:**